# A commentary on ‘Predicting recurrence and survival in patients with non-metastatic renal-cell carcinoma after nephrectomy: a prospective population-based study with multicenter validation’

**DOI:** 10.1097/JS9.0000000000001349

**Published:** 2024-03-27

**Authors:** Aimin Jiang, Peng Luo, Chenxin Chen, Chen Cai, Linhui Wang

**Affiliations:** a Department of Urology, Changhai Hospital, Naval Medical University; b Department of Special Clinic, Changhai Hospital, Naval Medical University; c Department of Health Statistics, Naval Medical University, Shanghai; d Department of Oncology, Zhujiang Hospital, Southern Medical University, Guangzhou, People’s Republic of China

*Dear Editor,*

Renal cell carcinoma (RCC) ranks among the top 10 most prevalent cancers in both genders and is projected to have caused 81 610 new cases in the United States in 2023^1^. The assessment of tumor recurrence risk in patients with localized RCC post-surgery commonly relies on TNM stage and pathological grade. Nevertheless, individuals with identical TNM stage and pathological grade may exhibit varying outcomes, indicating that the existing staging system lacks adequate precision in predicting tumor recurrence and advising on the choice of adjuvant therapy. In light of these considerations, we have taken a keen interest in the recent publication in the *International Journal of Surgery* entitled ‘Predicting recurrence and survival in patients with non-metastatic renal-cell carcinoma after nephrectomy: a prospective population-based study with multicenter validation’ by Wang *et al*.^2^ This study examines the prediction of recurrence and survival in patients with non-metastatic RCC following nephrectomy, utilizing a prospective population-based cohort for model construction and multicenter data for validation. The research delineates the creation and verification of prognostic models integrating standard clinicopathologic variables, exhibiting enhanced predictive efficacy relative to current models. Utilizing data from a substantial cohort in China and cross-validated with a U.S.-based cohort, the study underscores the durability, precision, and prospective clinical applicability of the models across various RCC histologies. This study demonstrates notable clinical value; however, certain conclusions presented in the article warrant further contemplation. We anticipate the authors’ elucidation and refinement of these findings in forthcoming research.

First, in the context of RCC progression, locally advanced RCC is characterized by tumor invasion beyond the renal capsule, encompassing perinephric or renal sinus fat, while remaining within the renal fascia, potentially accompanied by regional lymph node metastasis and/or venous tumor thrombus, without distant metastasis (T1-2N1M0/T3N0-1M0 stages). Furthermore, in the absence of distant metastasis, extension beyond the renal fascia, such as adrenal invasion or involvement of adjacent organs (T4), also qualifies as locally advanced RCC. The perioperative management of locally advanced RCC continues to be a significant focus of ongoing clinical investigation^3^. The progress in pharmaceutical trials and advancements in surgical equipment have greatly increased the success rates of surgeries, thereby improving the prognosis for patients. In the research conducted by Wang *et al*., it was observed that locally advanced RCC patients represented 14% and 24% in the developing and validation sets, respectively (Table 1). Nevertheless, the specific neoadjuvant treatments administered to these patients and the distribution of treatments were not specified. Due to the significant attention garnered by numerous large-scale, randomized controlled trials investigating neoadjuvant therapies such as stereotactic ablative radiotherapy, targeted therapy, and immunotherapy, there is a need for additional examination of the generalizability of prognostic model findings to patients undergoing neoadjuvant treatments.

Second, we noted that the cohort for this study consisted of RCC patients who received surgical intervention at the institution from 2006 to 2022. It is important to highlight that the percentage of patients treated during the 2019 coronavirus pandemic within this group was not explicitly stated. It is worth mentioning that ACE2 and TMPRSS2 demonstrate elevated expression levels in podocytes and proximal renal tubule cells, underscoring the kidney’s significance as a crucial target organ for SARS-CoV (severe acute respiratory syndrome coronavirus) infection^4^. Several clinical cohort studies have concurrently documented a notable rise in postoperative risks among renal cancer patients undergoing surgery amidst the pandemic, suggesting that the omission of such information could potentially influence the conclusions of this study^3^.

Furthermore, despite undergoing validation through internal and external methods, as well as adjustments for histology and year of surgery in clinicopathological analysis, the entry and exit criteria of forward and backward selection techniques seem overly strict. This inflexibility may result in the exclusion of important clinical features that should be included in the model. Properly relaxing the criteria could improve the variable selection process. The implementation of advanced variable selection techniques, such as machine learning, presents a potential opportunity for enhancing the quality of data analysis by addressing issues associated with collinearity. Additionally, it was noted that Wang *et al*.^5^ managed missing values through a case-by-case deletion approach. However, despite the presence of a small proportion (<10%) of missing values in certain variables, exclusively utilizing complete cases for model development may lead to biased results and overlook important patient characteristics, particularly those with incomplete clinical data. The utilization of data imputation techniques has the potential to improve the efficacy of prognosis models. The comprehensive exploration of various variable selection strategies and data imputation methods through sensitivity analyses could further enhance the study’s depth and breadth.

Last, despite the reliance on clinical indices for the prognostic parameters in this study, it is evident that the development of multimodal analysis scoring systems for predicting outcomes in RCC has been a notable area of research. Recent studies have incorporated deep learning models, genetic information, and imaging data to create more refined predictive tools. An example of this is a study conducted by Gui *et al*., in which they created and verified a new scoring system that combines clinical, genomic, and histopathological data to enhance the predictive precision of localized RCC recurrence. This scoring system accurately forecasted the recurrence-free period of patients in the training group and two validation sets, with respective areas under the curve at 5 years ranging from 0.825 to 0.876 and 0.608 to 0.793^6^. Therefore, the incorporation of deep learning models, genetic information, imaging data, and clinical parameters presents a potentially effective method for enhancing the clinical management of RCC, facilitating the development of personalized and efficient treatment plans.

In general, this study emphasizes the significance of enhancing prognostic modeling in RCC to enhance outcomes and individualize care, thereby laying the groundwork for novel research avenues in the field of oncology.

## Ethical approval

Not applicable.

## Consent

Not applicable.

## Sources of funding

Not applicable.

## Author contribution

A.J., P.L., and ChenxinC.: data collection and manuscript preparation; ChenC. and L.W.: study concept or design and critical revision and submission.

## Conflicts of interest disclosure

There are no conflicts of interest.

## Research registration unique identifying number (UIN)

Not applicable.

## Guarantor

Linhui Wang.

## Data availability statement

The datasets generated and/or analyzed during the current study are available from the corresponding author on reasonable request.

## Provenance and peer review

Not applicable.
